# Impact of long-term elosulfase alfa treatment on respiratory function in patients with Morquio A syndrome

**DOI:** 10.1007/s10545-016-9973-6

**Published:** 2016-08-23

**Authors:** Christian J. Hendriksz, Kenneth I. Berger, Rossella Parini, Moeenaldeen D. AlSayed, Julian Raiman, Roberto Giugliani, John J. Mitchell, Barbara K. Burton, Norberto Guelbert, Fiona Stewart, Derralynn A. Hughes, Robert Matousek, Elaina Jurecki, Celeste Decker, Paul R. Harmatz

**Affiliations:** 1 The Mark Holland Metabolic Unit, Salford Royal NHS Foundation Trust, Ladywell NW2- 2nd Floor Room 112 Salford, Manchester, M6 8HD UK; 2 New York University School of Medicine, André Cournand Pulmonary Physiology Laboratory, Bellevue Hospital, New York, NY USA; 3 Azienda Ospedaliera San Gerardo, Monza, Italy; 4 King Faisal Specialist Hospital & Research Center, Riyadh, Saudi Arabia; 5 Hospital for Sick Children, Toronto, ON Canada; 6 Med Genet Serv HCPA, Dep Genet UFRGS & INAGEMP, Porto Alegre, Brazil; 7 Montreal Children’s Hospital, Montreal, QC Canada; 8 Lurie Children’s Hospital & NWU Feinberg, Chicago, IL USA; 9 Hospital de Niños de Cordoba, Cordoba, Argentina; 10 Belfast City Hospital, Belfast, NI UK; 11 Royal Free London NHS Foundation Trust & UC, London, UK; 12 BioMarin Pharmaceutical Inc., Novato, CA USA; 13 UCSF Benioff Children’s Hospital Oakland, Oakland, CA USA

## Abstract

**Objective:**

To present long-term respiratory function outcomes from an open-label, multi-center, phase 3 extension study (MOR-005) of elosulfase alfa enzyme replacement therapy (ERT) in patients with Morquio A syndrome.

**Methods:**

In part 1 of MOR-005, patients initially randomized to ERT in the 24-week pivotal study (MOR-004) remained on their regimen (2.0 mg/kg/week or every other week); placebo patients were re-randomized to one of the two regimens. During part 2, all patients received elosulfase alfa 2.0 mg/kg/week. Respiratory function was one of the efficacy endpoints evaluated in MOR-005. Change from MOR-004 baseline to 120 weeks of treatment for the combined population was determined and compared with results from untreated patients from a Morquio A natural history study (MorCAP).

**Results:**

Maximum voluntary ventilation (MVV) improved up to week 72 and then stabilized; forced vital capacity (FVC) and forced expiratory volume in 1 s (FEV_1_) increased continuously over 120 weeks. Mean increases in the modified per-protocol population was 9.2 % for FVC, 8.8 % for FEV_1_, and 6.1 % for MVV after 120 weeks. All patients ≤14 years showed respiratory improvements, presumably in part related to growth; however, these were greater in treated patients. For those >14 years, treated patients showed improvements, while deterioration occurred in untreated. Altogether, the improvements were significantly greater (*P* < 0.05) in treated patients.

**Conclusions:**

Long-term ERT is associated with sustained improvements in respiratory function in Morquio A. In younger patients (≤14 years), some improvement may be ascribed to growth. In older patients, other mechanisms, e.g., decreased glycosaminoglycan storage, are likely involved.

**Electronic supplementary material:**

The online version of this article (doi:10.1007/s10545-016-9973-6) contains supplementary material, which is available to authorized users.

## Introduction

Morquio A syndrome (mucopolysaccharidosis (MPS) IVA; OMIM #253000) is a rare inherited lysosomal storage disorder in which deficiency of the lysosomal enzyme *N*-acetylgalactosamine-6-sulfatase (GALNS, EC 3.1.6.4) causes progressive accumulation of the glycosaminoglycans (GAGs) chondroitin-6-sulfate and keratan sulfate (KS) in tissues and organs. This accumulation can lead to musculoskeletal abnormalities, short trunk, dysfunctions in the respiratory, cardiac, neurological, and gastrointestinal systems, impaired vision, and hearing loss (Harmatz et al 2013; Hendriksz et al 2013).

Respiratory manifestations are among the most common causes of mortality in Morquio A patients (Lavery and Hendriksz 2015) and include upper and lower airway obstruction and restrictive pulmonary disease (Berger et al 2013). Airway obstruction can develop due to GAG accumulation in upper and lower airways and secondary to inflammation, abnormalities of the spine (e.g., short neck, high epiglottis, abnormal cervical vertebrae), and abnormalities of trachea and main stem bronchii (Simmons et al 2005; Sims and Kempiners 2007). Restrictive disease can be due to a reduction of lung volume secondary to short stature and thoracic cage deformities or impaired motility of the diaphragm due to liver enlargement or respiratory muscle weakness (Berger et al 2013; Harmatz et al 2013; Hendriksz et al 2013). Spinal cord compression and cardiac disease may also cause or aggravate respiratory problems (Berger et al 2013). In an early stage, obstructive and restrictive disease can lead to recurrent airway infections and sleep disordered breathing (Leighton et al 2001; Muhlebach et al 2011). Over time, they may lead to daytime hypoventilation and eventual respiratory failure (Pelley et al 2007). Respiratory impairment also considerably increases the risk of complications with anesthesia during surgery (Walker et al 2013).

Currently, the only approved therapy for Morquio A is elosulfase alfa (Vimizim®, BioMarin Pharmaceutical Inc., Novato, CA) enzyme replacement therapy (ERT) (Sanford and Lo 2014). The efficacy and safety of this therapy has been established in a 24-week, double blind, randomized, placebo-controlled, phase 3 study including 176 Morquio A patients (MOR-004) (Hendriksz et al 2014, 2015). After 24 weeks of treatment with 2.0 mg/kg/week elosulfase alfa, patients showed a significant improvement in the 6-min walk test (6MWT) distance, a numerical improvement in the 3-min stair climb test (3MSCT), and a significant decline in urine KS (Hendriksz et al 2014, 2015). The study also showed numerical improvements in respiratory function tests. At 24 weeks, maximum voluntary ventilation (MVV), forced vital capacity (FVC), and forced expiratory volume in one second (FEV_1_) increased by a mean of 10.3, 3.3, and 1.8 %, respectively, compared to placebo (Hendriksz et al 2014).

Evaluation of results from the long-term, open-label extension of MOR-004 (MOR-005) showed an acceptable safety profile consistent with MOR-004, sustained urine KS reduction, and sustained improvements in endurance over 2 years in treated patients when compared to corresponding untreated patients from the Morquio A Clinical Assessment Program natural history study (MorCAP, #NCT00787995) (Hendriksz et al 2016). Here, we present the respiratory function outcomes after 120 weeks of treatment with elosulfase alfa in the MOR-005 study.

## Methods

### Study design

**Fig. 1:**
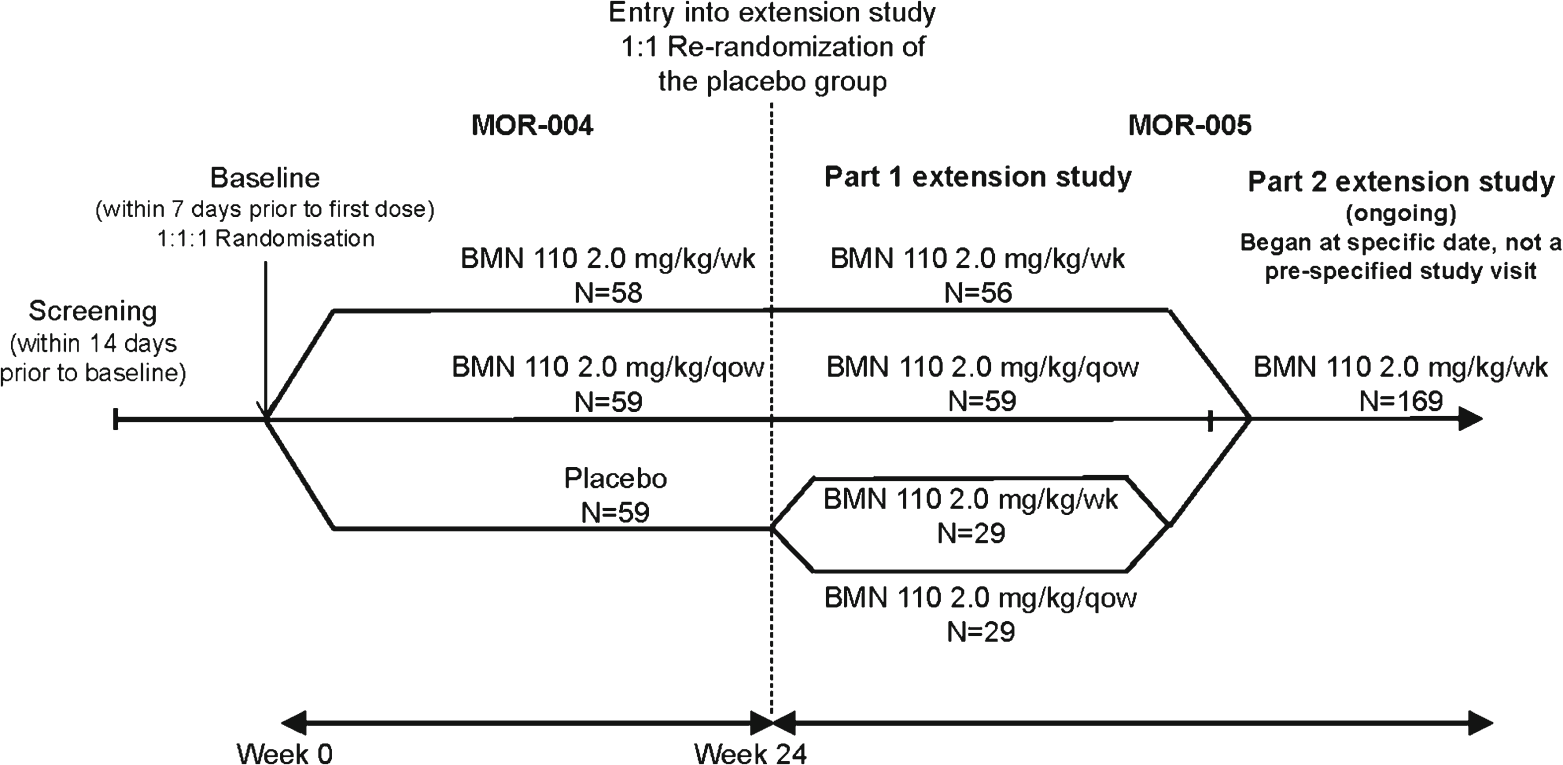
Study design of MOR-004 and MOR-005. Of the 176 patients enrolled in MOR-004, 175 completed the MOR-004 and one withdrew after a single infusion. Of these, 173 patients continued into MOR-005 and two declined to provide informed consent for the extension study

MOR-005 (#NCT01415427) is a multi-national, multi-center, open-label, phase 3, extension of the randomized, double-blind, placebo-controlled, 24-week phase 3 study (MOR-004; #NTC01275066). The 24-week pivotal study included 176 Morquio A patients ≥5 years of age (Hendriksz et al 2014, 2015). All patients completing the pivotal study were eligible for enrolment in the extension study. The study design of MOR-004 and MOR-005 (Fig. 1) has been previously discussed in detail (Hendriksz et al 2014, 2016). MOR-005 consisted of two parts. In part 1, patients initially randomized to elosulfase alfa during MOR-004 remained on their assigned dosing regimen of 2.0 mg/kg/week or every other week (qow). Placebo-treated patients were re-randomized (1:1) to one of the two dosing regimens. Part 2 started at a specific date when all patients were switched to elosulfase alfa 2.0 mg/kg/week, which was established as the recommended dose after review of the final results of MOR-004. Timing of transition to weekly dosing depended on study enrolment timing and ranged from week 36 to 96.

### Evaluation of respiratory function

The respiratory function efficacy evaluations conducted in this extension study are identical to those conducted during the 24-week pivotal trial, which have been described in detail previously (Hendriksz et al 2014). Respiratory function was assessed as one of the measures of long-term efficacy and tests included FVC, FEV_1_, and MVV. These were performed every 24 weeks in part 1 of MOR-005 and every 48 weeks in part 2. All respiratory function tests were conducted in accordance with the American Thoracic Society standards (American Thoracic society 1995).

Due to the long-term nature of this study, a placebo group was not included. Therefore, respiratory function data from a comparable untreated patient population from the multi-center, cross-sectional MorCAP natural history study (Harmatz et al 2013, 2015) were used to place the results in the context of a progressive disease. MorCAP patients included in the comparisons with the MOR-005 modified per-protocol (MPP, see statistical methods for explanation) population met the inclusion criteria for MOR-005 (≥5 years of age; average 6MWT distance ≥30 and ≤325 m at baseline), had longitudinal data (year 1 and/or year 2) available, and reported no major surgeries during the relevant time period of 2 years.

### Statistical methods

The analyzed data were collected during the 24-week MOR-004 study and 96 weeks of the MOR-005 extension study, representing a total of up to 120 weeks of ERT (96 weeks for the patients originally receiving placebo). Results are reported for the intent-to-treat (ITT) and MPP population. The ITT population includes all patients who were previously included in the 24-week MOR-004 study and had received at least one dose of elosulfase alfa in MOR-005; the MPP population (a subset of the ITT population) excludes patients who had an orthopedic surgery during the study or exhibited non-compliance (defined as missing ≥20 % of scheduled infusions). Further details are described in the primary MOR-005 publication (Hendriksz et al 2016). Descriptive statistics of respiratory function endpoints are provided for both populations.

The variable timing of transition to weekly dosing (from week 36 to week 96) precluded comparison of dosing regimens. However this decision was considered to be in the best interest of the patients. In addition, comparisons were also limited by the small sample sizes of the two treatment groups originally randomized to placebo. Therefore, all groups, representing patients consistently treated with the more optimal weekly dosing as well as patients that received qow dosing, were combined for the analysis.

A repeated measures analysis of covariance (ANCOVA) model was used to compare least square mean (LS mean) changes from baseline at year 1 and 2 of the MOR-005 MPP and MorCAP populations. The model included treatment, time point, baseline height, treatment and time point interaction, age group, and baseline measurement. Correlations between change in FVC and change in height were estimated using the Pearson correlation coefficient (r).

## Results

### Patient characteristics

Of the 175 patients who completed the 24-week pivotal study (MOR-004), 173 enrolled in the MOR-005 extension study (Hendriksz et al 2016). Of the 353 untreated patients in MorCAP, 79 met the inclusion criteria for comparison with the MOR-005 MPP population. The exact time of follow-up visits in MorCAP varied; visit windows ranged from approximately 38 to 87 weeks (mean follow-up 63.7 weeks or 446 days) for the year 1 evaluation and approximately 87 to 135 weeks (mean follow-up 107 weeks or 749 days) for the year 2 evaluation. These were compared with the 72 and 120 week time points of MOR-004/MOR-005.

Supplementary Table 1 shows demographics and baseline characteristics of patients who entered MOR-005 and of the untreated patients from the MorCAP study used for comparison. For MorCAP patients, baseline data are shown for all patients contributing data at baseline and separately for patients contributing data at year 1 and/or year 2. Both patient populations had similar baseline characteristics (see Table 1 for respiratory function baseline values), regardless of the time point of comparison.

**Table 1:** Change from MOR-004 baseline in forced vital capacity (FVC), forced expiratory volume in 1 s (FEV_1_), and maximum voluntary ventilation (MVV) over 120 weeks in the MOR-005 intent-to-treat (ITT) and modified per-protocol (MPP) populations

	ITT				MPP			
	Baseline*	24 weeks	72 weeks	120 weeks	Baseline*	24 weeks	72 weeks	120 weeks
**FVC**, L	1.1 (0.7)				1.1 (0.8)			
Mean (SE) change from baseline		0.025 (0.009)	0.054 (0.012)	0.076 (0.018)		0.024 (0.011)	0.062 (0.012)	0.087 (0.021)
Mean (SE) % change from baseline		3.5 (1.0)	7.4 (1.3)	8.6 (1.7)		3.7 (1.2)	7.9 (1.4)	9.2 (1.9)
**FEV**_**1**_, L	0.9 (0.6)				1.0 (0.6)			
Mean (SE) change from baseline		0.019 (0.009)	0.039 (0.011)	0.053 (0.017)		0.017 (0.011)	0.044 (0.011)	0.065 (0.019)
Mean (SE) % change from baseline		3.5 (1.2)	6.2 (1.5)	7.7 (2.1)		3.7 (1.4)	6.7 (1.7)	8.8 (2.3)
**MVV**, L/min	31.9 (21.8)				34.3 (23.5)			
Mean (SE) change from baseline		1.38 (0.60)	1.78 (0.74)	1.80 (1.04)		1.54 (0.75)	1.77 (0.89)	1.84 (1.21)
Mean (SE) % change from baseline		6.9 (1.9)	10.9 (2.7)	11.0 (4.6)		7.5 (2.3)	9.6 (2.7)	6.1 (3.2)

**Fig. 2:**
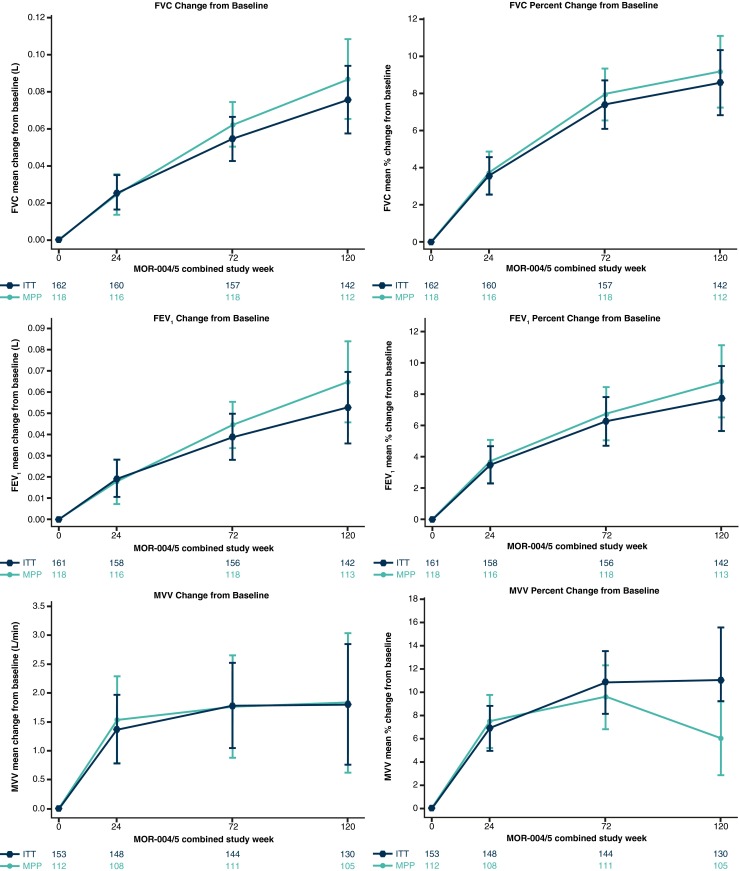
Respiratory function changes from MOR-004 baseline in the MOR-005 intent-to-treat (ITT; *dark-blue triangles*) and modified per-protocol (MPP; *green circles*) populations receiving long-term elosulfase alfa treatment. *Error bars* represent standard error; Ns are shown at the bottom of each graph

Ns are the same as those presented for each group in Fig. 2

*Baseline: mean (SD)

### Respiratory function over 120 weeks

During the 24-week pivotal study, numerical improvements in FVC, FEV_1_, and MVV were observed compared to placebo (Hendriksz et al 2014). During MOR-005, FVC and FEV_1_ continuously increased up to week 120 (Fig. 2). MVV continued to improve during the extension study up to week 72, and then stabilized by week 120 (Fig. 2). The mean (SE) FEV_1_/FVC ratio for the MOR-005 MPP population was 0.9 (0.01) at baseline and remained constant over time.

Figure 2 and Table 1 show changes in respiratory function outcomes from MOR-004 baseline over 120 weeks in the ITT and MPP populations.

### Respiratory function in treated patients versus untreated natural history controls

**Fig. 3:**
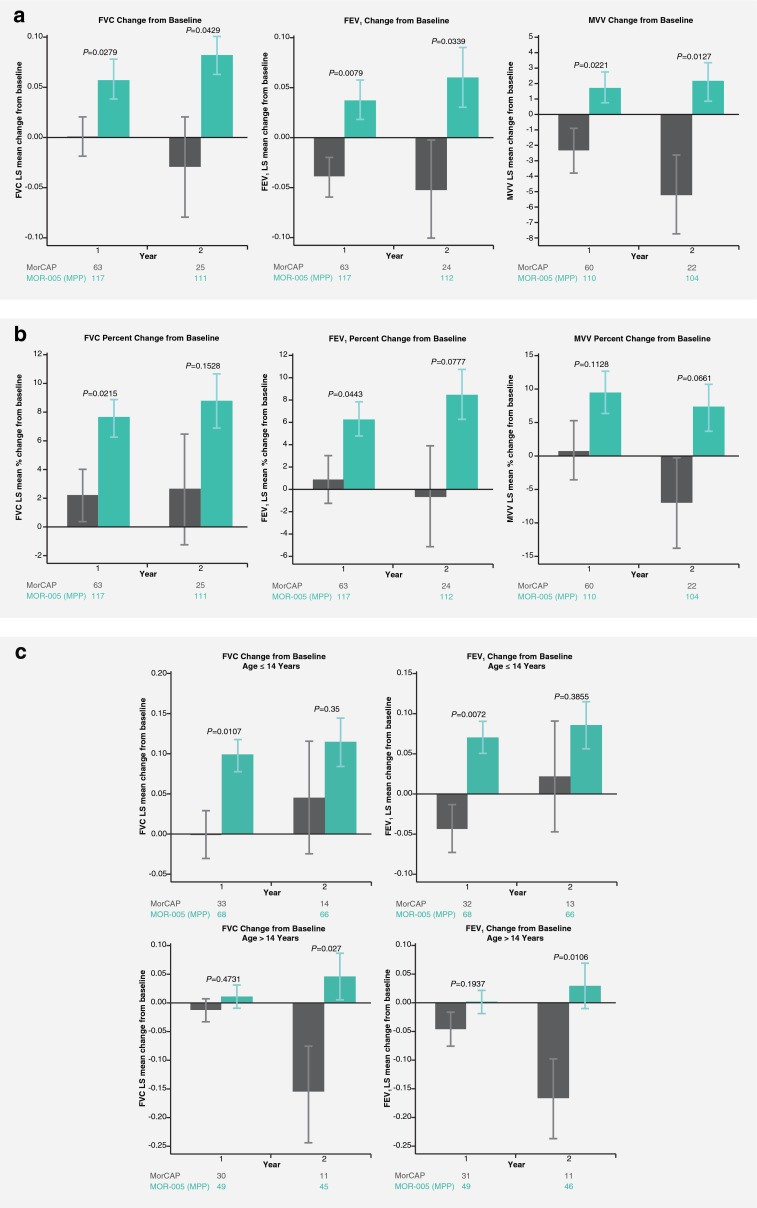
Least square (LS) mean respiratory function changes from MOR-004 baseline in the MOR-005 modified per-protocol (MPP; *green bars*) population treated with elosulfase alfa versus corresponding untreated patients from the MorCAP (gray bars) natural history study (ANCOVA analysis). **a** FVC, FEV_1_, and MVV LS mean change from baseline **b** FVC, FEV_1_, and MVV LS mean percent change from baseline **c** FVC and FEV_1_ LS mean change from baseline in patients aged ≤14 years and >14 years. Error bars represent standard error; Ns are shown at the bottom of each graph

ANCOVA analysis demonstrated significant improvements from baseline in the MOR-005 MPP population versus corresponding untreated patients from the MorCAP natural history study for FVC, FEV_1_, and MVV at 1 and 2 years (*P* < 0.05) (Fig. 3a). A similar, though not consistently significant, trend was seen for percent changes from baseline (Fig. 3b). LS mean changes from MOR-004 baseline in FVC were +0.0589 L (7.6 %) and +0.0827 L (8.8 %) in MOR-005 versus +0.0008 L (2.2 %) and −0.0299 L (2.6 %) in MorCAP at years 1 and 2, respectively. LS mean changes in FEV_1_ were +0.0385 L (6.3 %) and +0.06 L (8.5 %) in MOR-005 versus −0.0399 L (0.9 %) and -0.052 L (−0.6 %) in MorCAP at years 1 and 2, respectively. LS mean changes in MVV were +1.7 L/min (9.6 %) and +2.1 L/min (7.3 %) in MOR-005 versus −2.4 L/min (0.8 %) and −5.2 L/min (−7.0 %) in MorCAP at years 1 and 2, respectively. The mean (SE) FEV_1_/FVC ratio at baseline for MorCAP was the same as for MOR-005 MPP, 0.9 (0.01), and did not change over time.

ANCOVA analyses of FVC and FEV_1_ were also performed for patient subgroups ≤14 and >14 years, as growth is limited beyond the age of 14 in Morquio A patients (Harmatz et al 2015). Both FVC and FEV_1_ improved in MOR-005 MPP patients versus the untreated MorCAP population, regardless of age group (Fig. 3c). In the ≤14 years subgroup, both treated and untreated patients showed improvements, presumptively due to growth; however, the improvements were greater in treated patients. In this ≤14 years subgroup, the MOR-005 MPP patients (*N* = 67) had a mean change (SE) in height of +5.1 (3.5) cm compared to +2.8 (2.8) cm in the untreated MorCAP patients (*N* = 12). In the >14 years subgroup, treated patients showed an improvement in respiratory function while deterioration occurred in untreated patients. A difference was also observed in mean change (SE) in height, +1.3 (3.2) cm in the MOR-005 MPP (*N* = 43) patients compared to −0.0 (0.9) cm in the untreated MorCAP patients (*N* = 10). In younger patients, improvements in FVC correlated with increases in height (MOR-005 *r* = 0.32 and MorCAP *r* = 0.43; Supplementary Fig. 1A). However, in older patients, no correlation was evident (MOR-005 *r* = 0.14 and MorCAP *r* = −0.22; Supplementary Fig. 1B), suggesting involvement of other mechanisms.

## Discussion

ERT with elosulfase alfa is the only approved treatment for patients with Morquio A targeting the underlying cause of the disease, GALNS deficiency. Previously published data from MOR-005 have shown that long-term treatment with elosulfase alfa is associated with significant improvements in endurance measures and a decrease in urine KS for up to 120 weeks, when compared to untreated patients from the MorCAP natural history study (Hendriksz et al 2016). The results of the present analysis indicate that elosulfase alfa is associated with long-term sustained improvement in respiratory function as well.

MVV increased rapidly in the first 24 weeks and kept improving until 72 weeks, after which it stabilized. The positive trends in FVC and FEV_1_ observed during the pivotal study (Hendriksz et al 2014) continued during the extension study for up to 120 weeks, although at a slower initial improvement rate than MVV. This slower rate of improvement in FVC and FEV_1_ compared to MVV highlights that some pulmonary functions require longer periods of treatment to show improvement, similar to observations for MPS VI (Harmatz et al 2010).

The observed improvement in respiratory function over a 2-year period is remarkable considering the progressive nature of Morquio A, which leads to worsening of respiratory function with increasing age (Tomatsu et al 2011; Hendriksz et al 2013). The decline that is observed over a 2-year period in untreated patients underpins the significance of the improvements that are observed in this study. Given the morbidity and mortality associated with decreasing respiratory function in Morquio A (Lavery and Hendriksz 2015), the improvements reported here are particularly meaningful.

There was no placebo group included as continuing with weekly placebo infusions and withholding treatment for the duration of this long-term extension study was considered unethical. In the absence of a placebo group, the MOR-005 data were compared to that of a similar untreated Morquio A patient population from the MorCAP natural history study. The improvements from baseline in respiratory function outcomes in MOR-005 differed significantly from the gradual decline seen in the MorCAP population, suggesting that elosulfase alfa slows down, and even reverses the natural progression of respiratory dysfunction. The interpretation of the results was complicated by differences in timing of transition from elosulfase alfa qow to weekly dosing, which depended on study enrolment timing and ranged from week 36 to week 96. Therefore, a proportion of the patients in MOR-005 had only been on weekly dosing for 26 weeks. This may have biased analysis in favor of finding no effect of treatment since qow dosing was found to be suboptimal based on the pivotal MOR-004 trial results (Hendriksz et al 2016). Due to the staggered transition, the effect of long-term qow treatment alone could not be accurately assessed and any conclusions drawn would be speculative in nature. Limitations of the comparison with patients from the MorCAP study have been discussed previously and include the decreasing number of observations over time in the MorCAP population, potential differences that may exist between the MorCAP and MOR-005 populations and test executions, and the fact that some patients contribute to both populations (Hendriksz et al 2016). Despite this, baseline demographics and characteristics of the populations used in the analyses were relatively similar.

FVC, FEV_1_, and MVV assessments are physically demanding tests as they require maximal effort, which can be challenging for MPS patients that have limited lung capacity due to their short stature, chest wall deformities, respiratory muscle weakness, and upper airway obstruction (Berger et al 2013; Harmatz et al 2013; Hendriksz et al 2013). The observed increase in respiratory function in the MOR-005 population may be directly or indirectly attributed to several mechanical, physiological, and/or anatomical factors. As lung volume is related to body size, growth may be an important factor in younger patients. The small increases in FVC and FEV_1_ over 2 years seen in untreated MorCAP patients ≤14 years old are likely due, in large part, to growth. Improvements in FVC correlated with increases in height in these patients. Increases in FVC and FEV_1_ were greater in treated patients than in untreated patients. This may be caused in part by ERT-induced growth acceleration, as treated patients showed an increase in height of +5.1 cm over 2 years compared to only +2.8 cm in the untreated patients. However, FVC and FEV_1_ also improved in ERT-treated patients >14 years, while the untreated MorCAP patients in this age group showed considerable decline. Growth was very limited in older patients (+1.3 cm in treated vs -0.0 cm in untreated) suggesting that the increase in respiratory function by ERT was most likely mediated by other mechanisms, such as decreased upper airway obstruction, increased chest wall compliance, improved respiratory muscle strength, and/or improved diaphragmatic movement due to a reduction in liver size and a declined GAG tissue storage (Harmatz et al 2010). These mechanisms may, in addition to growth, also have contributed to the improved respiratory function in younger patients.

Compared with FVC and FEV_1_, MVV increased relatively rapidly in our study, particularly during the first 24 weeks, but stabilized after 72 weeks. A similar pattern of early response has been reported for MPS VI patients treated with ERT (Harmatz et al 2010). Even though MVV generally shows a good correlation with FEV_1_ (Pellegrino et al 2005), in patients with neuromuscular disease or upper airway obstruction a disproportional change in MVV relative to FEV_1_ has been observed (Engström et al 1964; Serisier et al 1982; Braun et al 1983). One possible explanation for our results is that ERT reduced airway obstruction. However, that is not supported by the observation that FEV_1_/FVC ratios remained stable throughout the study, whereas a reduced FEV_1_/FVC ratio suggests obstructive disease (Pellegrino et al 2005; Berger et al 2013). Therefore, the most probable cause of respiratory dysfunction in the study population is restrictive lung disease. This respiratory limitation is likely not only due to a low FVC, caused by the severe skeletal involvement in Morquio A (Muhlebach et al 2011; Harmatz et al 2013), but also the inability to increase respiratory rate due to muscle weakness, pain, and joint constriction. Improvement in respiratory rate can be attributed to both modestly improved FVC and FEV_1_ but also to improved MVV after ERT treatment.

Improvement and long-term stabilization of respiratory function with ERT has already been described for MPS I, II, and VI (Wraith et al 2004; Muenzer et al 2006, 2011; Clarke et al 2009; Harmatz et al 2010; Lin et al 2014). These results, together with the results of this study, suggest that the positive effect of ERT on respiratory function in MPS patients is probably caused by a variety of mechanisms associated with a decrease in GAG storage, including improvement of joint mobility, reduction in liver volume, improvement in endurance and respiratory muscle strength (Wraith et al 2004; Clarke et al 2009; Harmatz et al 2010; Muenzer et al 2011; Hendriksz et al 2016), reduced local inflammation, and an improvement of lung parenchyma (Yasuda et al 2013). All these mechanisms can contribute to improved neuromuscular function, leading to enhanced inspiratory effort and thus decreased pulmonary restriction.

## Conclusions

Impaired respiratory function is one of the leading causes of morbidity and mortality in Morquio A patients. The present study suggests that ERT with elosulfase alfa slows down, and partially reverses, the natural progression of respiratory dysfunction associated with Morquio A over a 2 year period. ERT-induced growth acceleration may considerably contribute to this effect in younger patients; it is likely that other mechanisms, related to decreasing GAG accumulation, also play a role in older patients. Over time, these improvements in respiratory function may lead to reductions in morbidity and mortality in Morquio A patients.

## Electronic supplementary material

Below is the link to the electronic supplementary material.

ESM 1 (DOC 80 kb)

ESM 2 (DOC 119 kb)
