# Supplementary material for: Impact of long-term elosulfase alfa treatment on respiratory function in patients with Morquio A syndrome
Source: J Inherit Metab Dis. 2016 Aug 23;39(6):839–47. doi: 10.1007/s10545-016-9973-6 (PMC5065598; doi:10.1007/s10545-016-9973-6)
Supplement: Supplementary file 1 — (DOC 80 kb) [file 10545_2016_9973_MOESM1_ESM.doc]

**Supplementary Table 1.** Demographics and baseline characteristics.

|  | **MOR-005**  **ITT** | **MOR-005**  **MPP** | **MorCAP subpopulation contributing data at** | | |
| --- | --- | --- | --- | --- | --- |
|  |  |  | **Baseline** | **Year 1** | **Year 2** |
| **Age at enrolment,** N | 173 | 124 | 79 | 63 | 25 |
| Median (range), yrs | 11.7 (5.0, 57.4) | 12.2 (5.0, 49.1) | 12.0 (5.0, 65.0) | 13.4 (5.5, 49.2) | 12.3 (5.0, 65.6) |
| **Sex,** N | 173 | 124 | 79 | 63 | 25 |
| Female, N (%) | 87 (50.3) | 58 (46.8) | 48 (60.8) | 39 (61.9) | 12 (48.0) |
| **6MWT,** N | 173 | 124 | 79 | 63 | 25 |
| Mean (SD), m | 209.5 (74.0) | 201.6 (74.9) | 210.4 (83.4) | 204.4 (83.4) | 214.4 (75.3) |
| **FVC,** N | 162 | 118 | 75 | 60 | 23 |
| Mean (SD), L | 1.1 (0.7) | 1.1 (0.8) | 1.1 (0.7) | 1.2 (0.7) | 1.2 (0.7) |
| **FEV_1_,** N | 161 | 118 | 74 | 60 | 21 |
| Mean (SD), L | 0.9 (0.6) | 1.0 (0.6) | 1.0 (0.6) | 1.0 (0.7) | 1.1 (0.5) |
| **MVV,** N | 153 | 112 | 69 | 52 | 21 |
| Mean (SD), L/min | 31.9 (21.8) | 34.3 (23.5) | 31.7 (17.7) | 33.6 (19.1) | 32.7 (16.3) |

6MWT: 6-minute walk test; FEV_1_: forced expiratory volume in 1 second; FVC: forced vital capacity; ITT: intent-to-treat; MVV: maximum voluntary ventilation; MPP: modified per-protocol
