# Supplementary material for: Impact of long-term elosulfase alfa treatment on respiratory function in patients with Morquio A syndrome
Source: J Inherit Metab Dis. 2016 Aug 23;39(6):839–47. doi: 10.1007/s10545-016-9973-6 (PMC5065598; doi:10.1007/s10545-016-9973-6)
Supplement: Supplementary file 2 — (DOC 119 kb) [file 10545_2016_9973_MOESM2_ESM.doc]

**
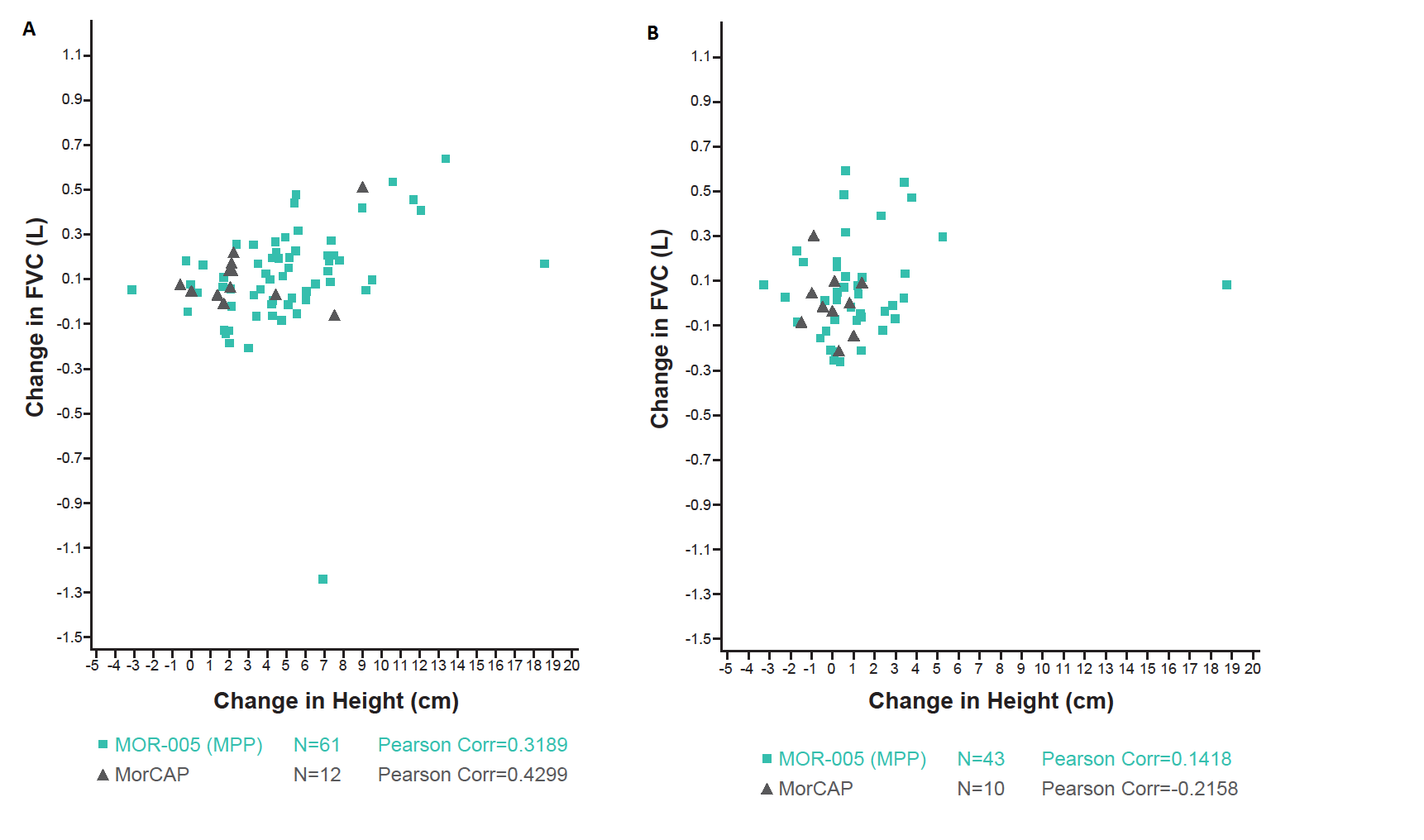
**

**Supplementary Figure 1.** Correlation between change in FVC and height at year 2 for patients in the MOR-005 and MorCAP MPP populations.

(A) Patients ≤14 years of age

(B) Patients >14 years of age
